# Pleural and Pericardial Infection Due to Cutibacterium acnes in a Splenectomized Patient: A Case Report of an Underreported Systemic Infection

**DOI:** 10.7759/cureus.13668

**Published:** 2021-03-03

**Authors:** Rafael C Da Silva, Onoriode Kesiena, Shreyas Singireddy, Jennifer Madeo

**Affiliations:** 1 Internal Medicine, Piedmont Athens Regional Medical Center, Athens, USA; 2 Infectious Disease, Piedmont Athens Regional Medical Center, Athens, USA

**Keywords:** pericarditis, cutibacterium, cardiac tamponade, propionibacterium, pericardiectomy

## Abstract

Infectious pericarditis does not always present with all the classic findings. Some of the traditional signs of fever, pleuritic chest pain, and frictional rub may be missing. This presents a diagnostic challenge, thus clinical suspicion is important. The most common cause of infectious pericarditis is viral. However, bacterial pericarditis may occur with severe complications such as constrictive pericarditis, pericardial effusion, cardiac tamponade, left ventricular pseudoaneurysm, and aortic mycotic aneurysm. The purpose of this presentation is to increase awareness of *Cutibacterium acnes* (*C. acnes) *as a cause of bacterial pericarditis. This case report highlights *C. acnes* as a prevalent cause of both pleural and pericardial infections. The diagnosis can be challenging, considering that this bacterium is difficult to isolate, slow growing, and causes indolent illness. Prolonged incubation time may be required. In addition to the more traditional causes of bacterial pericarditis, namely S*taphylococcus* and S*treptococcus *species, *C acnes* appears to play an important role. It should not be considered a contaminant as it may require further investigation.

## Introduction

Infectious pericarditis presents a diagnostic challenge in an immunocompromised patient due to poor inflammatory response. In this scenario, fever, pleuritic chest pain, and frictional rub may be missing [1,2]. The most common cause of infectious pericarditis is viral. However, bacterial pericarditis does occur with severe complications such as constrictive pericarditis, pericardial effusion, cardiac tamponade, left ventricular pseudoaneurysm, and aortic mycotic aneurysm [1,3]. Expected sources include contiguous spread from surrounding structures, direct inoculation from trauma involving the pericardium, cardiothoracic surgery, and hematogenous spread [1]. We report a rare case of pericarditis with concomitant pleural space infection due to *Cutibacterium acnes* (*C. acnes*) in a 54-year-old female with a previous history of splenectomy due to Hodgkin’s lymphoma. The goal of this presentation is to increase awareness of this mycroorganism as a cause of bacterial pericarditis.

## Case presentation

A 54-year-old female Caucasian patient with past medical history of paroxysmal atrial fibrillation, heart failure with preserved ejection fraction, Hodgkin’s lymphoma in remission, rheumatoid arthritis, and splenectomy was admitted to our hospital with one week of malaise, fever dry cough and shortness of breath. She also noted abdominal bloating and bilateral leg swelling. She denied chest pain. There was no history of trauma or recent surgery. At the time, she was on digoxin, diltiazem, and furosemide. Her vaccines were up to date. She had no history of cigarrete smoking, alcohol drinking comsuption or recreational drug use. She reported severe allergic reaction to penicillin. Physical examination on admission showed a frail middle-aged woman in no acute distress. Blood pressure 128/90 mmHg, pulse 130 irregular beats/min, respiratory rate 20 breaths/min, axillary temperature 97.6˚ Fahrenheit and oxygen saturation of 96% on room air. Body mass index was 16 kg/m2. Cardiovascular exam was remarkable for normal S1 and S2 and irregular rhythm. No murmurs or gallops were appreciated. Jugular venous distension was noticed. Pulmonary auscultation was remarkable for decreased breath sounds at the lung bases. The abdomen was mildly distended and there was hepatomegaly on palpation. Bilateral pitting edema 2+ was observed in the lower extremities. White blood count was 9500 cells/ul, hemoglobin 13,6 g/dl, blood urea nitrogen 22 mg/dl, creatinine 0,64 mg/dl, B-type natriuretic peptide 144 pg/ml, troponins negative and electrolytes within normal range.

**Figure 1:**
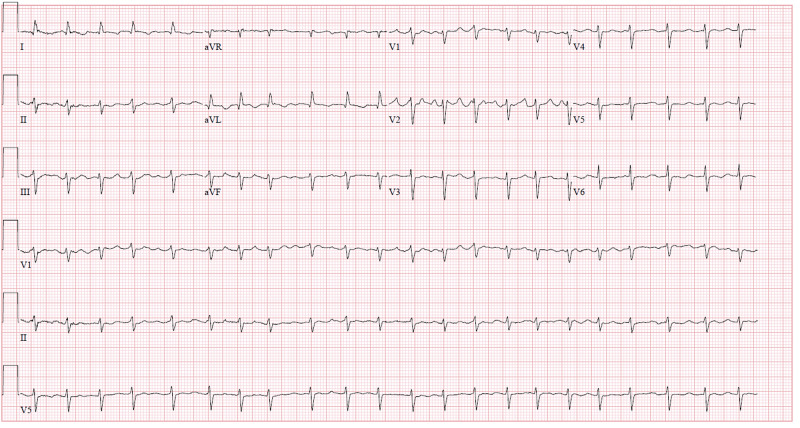
Electrocardiogram on admission. Note atrial fibrillation with rapid ventricular response and low voltage QRS in all leads (less than 5 mm in the limb leads and less than 10 mm in precordial leads). Left anterior fascicular block also noted.

**Figure 2:**
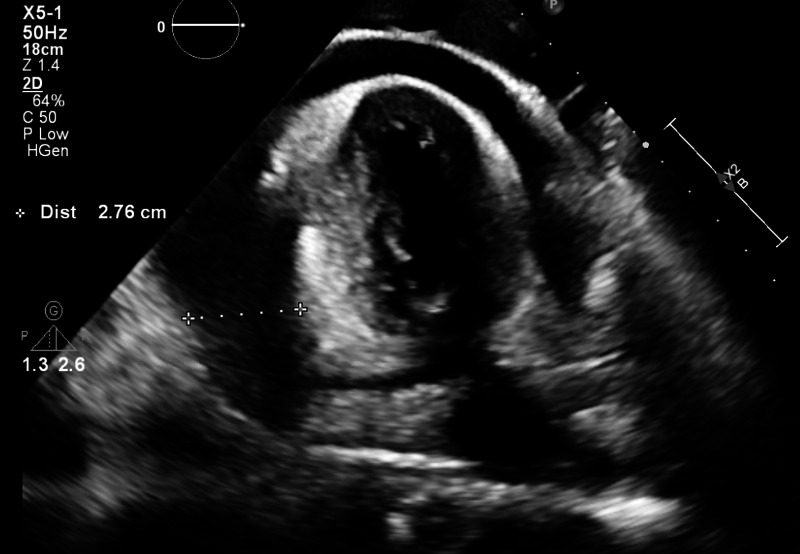
Transthoracic echocardiogram showed normal left ventricular function and large circumferential pericardial effusion.

Electrocardiogram (EKG) revealed atrial fibrillation with rapid ventricular response and generalized low voltage QRS (Figure 1). Chest X-ray showed bilateral moderate-sized pleural effusions with evident blunting of both costophrenic angles. Cardiomegaly was noted. CT of the chest showed moderate pericardial effusion, moderate right pleural effusion and small left pleural effusion, without evidence of pulmonary embolism. CT of the abdomen was significant for hepatic edema and mild ascites. Doppler ultrasound of the lower extremities showed no signs of deep venous thrombosis. Transthoracic echocardiogram (TTE) showed normal left ventricular function and large circumferential pericardial effusion (Figure 2).

Patient underwent pericardial window and right chest tube placement. Pleural and pericardial fluid were sent for analysis. The results are summarized in the Table 1.

**Table 1:** Pleural and pericardial fluid analysis.

	Pleural	Pericardial
	Pleural	Pericardial
Volume	400 ml	500 ml
Aspect	yellowish	bloddy
Cellularity	314 cels/mm3	1013 cels/mm3
Differential	55% lymphocytes	53% lymphocytes
Glucose	150 mg/dl	
Protein	2,3 g/dl	
Cytology	negative for malignancy	negative for malignancy
Fungal culture	negative	negative
Viral culture	negative	negative
Acid fast bacilli	negative	negative
Gram stain	negative	negative
Bacterial culture	*Cutibacterium acnes* (after 7 days)	*Cutibacterium acnes* (after 7 days)

After seven days of incubation, both pleural and pericardial fluid grew *C. acnes*. Pericardial tissue histology showed pericarditis with fibrinous exudate and organization. Due to penicillin allergy, the patient was treated initially with ceftriaxone and transitioned to oral linezolid at discharge. After two weeks of linezolid, she developed vision loss. Due to concerns about drug-induced optic neuritis, linezolid was switched to doxycycline for more two weeks. Her symptoms eventually resolved. New echocardiogram two weeks later showed reduction of pericardial and pleural effusion. 

## Discussion

In an earlier review of the literature between 1889 and 1975, Klacsmann et al. found *Streptococcus pneumoniae* as the prevalent bacterial cause of infectious pericarditis prior to 1943 and *Staphylococcus aureus* as the most prevalent after 1943 [4]. However, according to recent data, *Cutibacterium acnes*, formerly, *Propionibacterium acnes*, appears to play an important role as a causative agent of pericarditis [1,3,5-8]. In a retrospective study done at Mayo Clinic, the three frequently identified causes of bacterial pericarditis were *C. acnes, Staphylococci spp, and Streptococci spp*, with *C. acnes* being the most prevalent. In this study, some of the predisposing factors were immunosuppression, post-cardiac surgery, pneumonia, dental procedures, and connective tissue disease [9].

*Cutibacterium acnes* is considered normal flora of the skin, oral cavity, large intestine, and conjunctiva. It is a slow-growing gram-positive anaerobe that can cause severe infections, including bacteremia [10], subdural empyema [11], endocarditis [12,13], prosthetic valve infection, and prosthetic shoulder joint infection [13]. This is due to its ability to form biofilm and survive in anaerobic conditions [2,10]. It had also been reported as a cause of pleural infection [14]. In one report, the mechanism of infection of the pleural space by *C. acnes* was unknown, but the lead hypothesis was that it may have occurred as a consequence of a prior thoracentesis or pleuropulmonary seeding from the lymph nodes in patients with an underlying disease [14]. Iseki et al. reported a case of *C.acnes* pericarditis with calcifications mimicking a pericardial tumor [15].

To the best of our knowledge, this is the first case report of *C. acnes* as the cause of simultaneous infections of the pleural and pericardial space. Our patient did not have risk factors such as a recent dental procedure, prior cardiac surgery, or an implanted device. However, she had a history of splenectomy secondary to Hodgkin’s lymphoma, heart failure, and rheumatoid arthritis. One hypothesis is that both fluids accumulated due to acute on chronic heart failure which then served as a medium for a *C. acnes*. Another hypothesis is that *C. acnes* may have been the primary reason for the effusions. 

Most *Cutibacterium spp* are susceptible to penicillin, carbapenems, cephalosporins, and vancomycin. The duration of treatment depends on the source. They are inherently resistant to metronidazole [10]. There is limited evidence of the duration of antimicrobial treatment. Due to biofilm and anaerobic growth, this microorganism is difficult to eradicate [10]. Table 2 is a list of the most commonly used antibiotics. The patient in this case report was initially treated with ceftriaxone, followed by oral linezolid and finally doxycycline to complete a total of four weeks. 

**Table 2:** Antibiotics used to treat Cutibacterium acnes, according to multiple studies. CT: cardiac tamponade.

	date	n˚ patients	age/sex	pleural effusion	antibiotic	Complication
Iseki et al. [15]	1999	1	68m	N	not reported	constrictive
Mesado et al. [8]		1	26m	N	amoxicillin-clavulanate	constrictive/CT
		1	31m	N	amoxicillin/penicillin	constrictive
	2013	1	38f	N	ceftriaxone/doxycycline	constrictive
		1	55m	Y	amoxicillin-clavulanate	constrictive
		1	72m	Y	ceftriaxone/minocycline	not reported
Cruz et al. [6]	2015	1	61m	Y	penicillin/beta-lactams	constrictive
Jensen et al. [7]	2017	1	75m	N	penicillin/beta-lactams	constrictive
Farhat-Sabet et al. [3]	2018	1	71m	Y	vancomycin	constrictive/CT
Fakhri et al. [1]	2020	1	20m	N	penicillin/beta-lactams	cardiac tamponade

## Conclusions

This case report highlights *C. acnes* as a potential cause of both pleural and pericardial space infections. The diagnosis can be challenging, considering that this bacterium is difficult to isolate, slow growing, and causes indolent illness. Prolonged incubation time may be required. In addition to the more traditional causes of bacterial pericarditis, namely S*taphylococcus* and S*treptococcus *species*, C acnes* appears to play an important role. It should not be considered the only contaminant and may require further investigation.
